# Barriers and facilitators of hypertension control among older adults living with hypertension: A mixed-methods study

**DOI:** 10.1371/journal.pgph.0007015

**Published:** 2026-08-20

**Authors:** Prabha Shrestha, Biraj Man Karmacharya, Jennifer Merrilees, Yunika Acharya, Indu Poudel, Kedar Marahatta, Victor Valour

**Affiliations:** 1 Department of Nursing, Kathmandu University School of Medical Sciences, Dhulikhel, Nepal; 2 Research and Development Division, Dhulikhel Hospital, Kathmandu University Hospital, Dhulikhel, Nepal; 3 Global Brain Health Institute, University of California San Francisco, California, United States of America; 4 Memory and Aging Center, Department of Neurology, University of California, San Francisco, California, United States of America; 5 World Health Organization, Country office for Nepal, Kathmandu, Nepal; PLOS: Public Library of Science, UNITED STATES OF AMERICA

## Abstract

Hypertension is a major public health challenge in Nepal, particularly among older adults, with nearly half affected and many unaware of their condition. This study explored barriers and facilitators of hypertension management among older adults in Nepal using a parallel mixed-methods design. The study was conducted in Dhulikhel Municipality between September 2024 and March 2025. The quantitative component included 197 older adults (≥60 years) with hypertension. Blood pressure and body mass index (BMI) were measured, medication adherence was assessed using the Hill-Bone Medication Adherence Scale, and multivariable logistic regression was used to identify factors associated with blood pressure control. The qualitative component explored barriers and facilitators using the Capability, Opportunity, Motivation–Behavior (COM-B) model. Seven in-depth interviews with older adults and two focus group discussions with caregivers were conducted using semi-structured guides. Audio recordings were transcribed verbatim and analyzed using a framework approach with inductive and deductive coding. Overall, 59.3% of participants had uncontrolled blood pressure. No significant associations were found between blood pressure control and age, sex, education, marital status, religion, living arrangement, comorbidities, medication adherence, or BMI. Qualitative findings identified barriers across all COM-B domains, including fatigue and forgetfulness (capability), limited social interaction, poor access to information, time constraints, and low family support (opportunity), and low motivation, stress, and anger (motivation). Facilitators included greater awareness, use of pill boxes, regular blood pressure monitoring, access to nearby health facilities, community programs, support from Female Community Health Volunteers (FCHVs) and family members, fear of complications, perceived benefits of treatment, and the desire to remain healthy for family. These findings highlight the need for behavior-centered, patient-focused hypertension management strategies that integrate pharmacological and lifestyle interventions. Applying the COM-B framework can support the development of sustainable, contextually appropriate interventions to improve hypertension management among older adults in Nepal.

## Background

Globally, around 1.28 billion adults between the ages of 30 and 79 years have hypertension, with the majority, about two-thirds, residing in Low and Middle Income countries (LMICs) [1]. Hypertension is a major modifiable risk factor for cardiovascular disease, stroke, dementia and premature death worldwide and its prevalence rises with age affecting over two-thirds of adults aged 60 years and above [1,2]. Despite advances in antihypertensive drugs, blood pressure control remains low, especially among older population [3,4].

In Nepal, the burden of hypertension is increasing, particularly among the aging population. National surveys report that 46% of women and 42% of men aged 60 and above are hypertensive, indicating a significant public health challenge highlighting the country’s demographic shift toward an aging society [5]. A systematic review from Nepal reported that only 45% among hypertensive patients were aware of their high blood pressure and just 44% had their blood pressure under control [6]. However, limited health literacy and barriers to healthcare access may contribute to the underestimation of these figures, suggesting that the true prevalence of hypertension could be even higher [7].

Beyond cardiovascular risks, persistent hypertension is also associated to cognitive decline and dementia [8–10]. Hypertension contributes to vascular damage in the brain, reduced cerebral perfusion, and neurodegenerative changes, increasing the risk of dementia including vascular and Alzheimer’s types by approximately 1.2 to 1.5 times [11–13]. Hypertension has been recognized as one of the key modifiable risk factors for dementia [14].

Managing hypertension in later life is particularly complex due to age-related physiological decline and comorbidities [15]. These challenges are further influenced by psychosocial and behavioral factors such as frailty, low health literacy, and inadequate social support [16]. Studies show that social support can directly improve medication adherence and indirectly promote it by enhancing health literacy, which is itself strongly associated with better self-care practices [17].

To better understand and address these behavioral influences, theoretical frameworks such as the Capability, Opportunity, Motivation-Behavior (COM-B) model have been increasingly applied in health research [18,19]. The COM-B model puts that behavior change depends on an individual’s capability (physical and psychological), opportunity (physical and social environment), and motivation (reflective and automatic processes), providing a comprehensive structure for exploring barriers and facilitators to health behaviors, including hypertension management.

However, in Nepal, limited research has utilized such behavioral models to explore these influences, particularly among older adults. Given the rising prevalence of hypertension and its intersection with dementia and functional vulnerability, there is a pressing need to understand the factors shaping hypertension management behaviors in this population. This study therefore assesses blood pressure control status and explores barriers and facilitators to hypertension management among older adults in Nepal using the COM-B model.

## Methods

### Ethics statement

The Nepal Health Research Council (IRC registration number: 113_2024/Ref no: 2132) approved this study, and the Kathmandu University Institutional Review Committee (KU-IRC: 165/24) also granted ethical approval. Dhulikhel municipality provided permission to conduct the study. We obtained written informed consent from all participants. If a participant could not provide a signature, we collected their thumbprint along with the signature of a witness, typically a family member.

### Study design

We conducted a parallel mixed method study among older adults living with hypertensions of Dhulikhel, Nepal. The quantitative component involved a cross-sectional assessment of blood pressure control, medication adherence, and body mass index (BMI) whereas the qualitative component explored barriers and facilitators of hypertension management.

### Participant recruitment and sampling strategy

For the quantitative component, the study included 197 older adults living with hypertensions aged 60 years and above residing in Dhulikhel Municipality, in line with the definition provided by the Senior Citizens Act, 2063 of Nepal, which classifies individuals aged 60 years or older as senior citizens [20]. The sample size was calculated using the single population proportion formula. We assumed a 95% confidence level, a margin of error of 7%, and an expected prevalence of hypertension of 41.7% among older adults in Nepal. The minimum required sample size was calculated to be 191. After adding a 10% allowance for non-response, the target sample size was 210. However, we were able to successfully reach and enroll 197 eligible participants in the final survey. We used the Healthy City Initiative dataset of Dhulikhel Municipality. This dataset, jointly developed and maintained by Dhulikhel Hospital and Dhulikhel Municipality, contains household-level information on socioeconomic characteristics, health behaviors, morbidity, and mental health of municipal residents to support early identification and management of non-communicable diseases. Permission to access and use the dataset was obtained from the Principal Investigator of the Healthy City Initiative and Dhulikhel Municipality. The dataset included 1,594 individuals with hypertension. Among them, 766 individuals were aged 60 years and above and fulfilled the study eligibility criteria. From this eligible subset, 197 participants were randomly selected and recruited into the study using Stata software.

Prior to data collection, the researcher (PS and YA) visited the municipality to discuss the study and survey procedures with local stakeholders. For quantitative work, research assistants (RAs) contacted selected participants via telephone to schedule home visits at a convenient time. If participants could not be reached after five phone call attempts, RAs coordinated with Female Community Health Volunteers (FCHVs) from the respective wards to establish contact. Patients who were deceased or unreachable through both telephone and FCHVs were excluded from the study. Additionally, we assessed cognitive status of the participants using Rowland Universal Dementia Assessment Scale (RUDAS) [18] and those who were found to be cognitively impaired (n = 7) were excluded from the study; however, their blood pressure was measured and was reported to their family members [21]. Home visits were then scheduled. During these visits, RAs explained the purpose and procedures of the study and obtained written informed consent from participants. For those unable to read and write, the information sheet was read aloud, and consent was documented via thumbprint, co-signed by a literate caregiver.

For qualitative work, we purposely selected participants to capture diverse perspectives on barriers and facilitators to manage hypertension. These included seven older adults living with hypertension with uncontrolled blood pressure. We conducted two FGD with 18 caregivers of older hypertensive patients. Interviews with patients and caregivers were conducted near their households. FCHVs played a vital role in helping the research team to identify and connect with both hypertensive patients and their caregivers. All participants were contacted in advance, and interviews were scheduled at their convenience. None of the approached participants declined to participate.

The sample size was guided by the principles of code wherein interviews ceased at saturation to the point at which no new themes or insights emerge from the data [22]. Interviews were concluded by discussing with the research team members, once it became evident that no further unique codes or information were being identified.

We interviewed eligible participants using a structured questionnaire and responses were directly recorded into an online form developed in KoBoToolbox (https://kf.kobotoolbox.org/).

### Measurement

Socio-demographic variables included were: age (in years), sex (Male/Female), Marital status (Married, Never married/ Widowed/ Divorced or separated) Educational level (Not able to read and write/ Basic education/ Secondary education/ Secondary education and above) Ethnicity (Brahmin/Chettri/ Janjati/ Dalit), Religion (Hindu/Non Hindu), Living arrangement (living with son and daughter in law/ Couples only/ Alone/ Living with daughter and son in law/ Living with other relatives)adapted from previously conducted national surveys in Nepal [7,23]. Medication adherence was assessed using the Hill-Bone Medication Adherence Scale, which was already translated into Nepali language for contextual relevance [24,25].

Trained Research Assistants (RA) measured participants’ blood pressure (BP) on the left arm following a ten-minute rest period in a seated position. BP readings were obtained using a Microlife automatic blood pressure monitor. Prior to measurement, participants were instructed to avoid caffeine, exercise, and smoking for at least 30 minutes and to empty their bladder. To ensure accuracy, both the participant and the observer remained silent during the rest and measurement periods. Participants were asked to remove any clothing covering the upper arm to allow proper cuff placement. Blood pressure was measured while the participant was seated comfortably in a chair with their back supported, feet flat on the floor, and back upright. Body weight was measured with participants wearing minimal clothing and no shoes using an Omron Model HBF-400 digital scale and recorded in kilograms. Scales were calibrated daily. Height was measured without shoes, with participants standing upright against a flat wall, using measuring tape accurate to the nearest 0.1 cm. Body Mass Index (BMI) was calculated by dividing weight in kilograms by the square of height in meters (kg/m^2^).

### Data collection tool and technique

We interviewed eligible older adults living with hypertension using a structured questionnaire, and responses were directly recorded into an online form developed in KoBoToolbox (https://kf.kobotoolbox.org/). We conducted FGDs and IDIs, using a semi-structured interview guide. The interview guideline developed and refined through team discussions and pilot testing. Between September 2024 and March 2025, three trained qualitative researchers (PS, YA, and IP), all native Nepali speakers with prior experience in qualitative data collection, conducted the interviews. All interviews were held in Nepali, in private and quiet settings to ensure participant comfort and confidentiality. Each session lasted between 30 to 60 minutes.

### Data management and analysis

For the quantitative component, frequencies and percentages were calculated for categorical variables whereas mean and standard deviation were calculated for continuous variables. The HB-MAS measures the frequency of non-adherent behaviors related to medication intake. Each item is rated on a 4-point Likert scale ranging from 1 (“none of the time”) to 4 (“all the time”), with higher scores indicating poorer adherence. The total adherence score was calculated by summing all item scores, yielding a possible range of 9–36. We later classified BMI based on WHO guidelines: underweight (BMI < 18.5), normal weight (BMI 18.5–24.9), overweight (BMI 25–29.9), and obese (BMI ≥ 30) [26]. We defined uncontrolled hypertension as those whose systolic BP/diastolic BP was more than or equal to 140/90 mmHg [1]. We used multivariable logistic regression to determine the factor associated with uncontrolled hypertension in Stata version 14.

**Fig 1:**
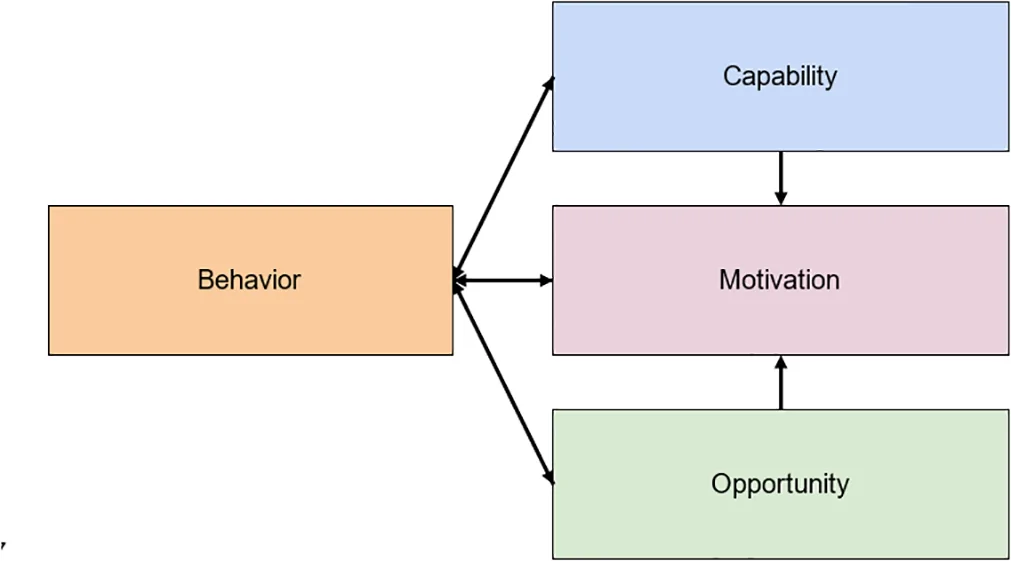
The COM-B model to explore the barriers and facilitators to hypertension management among older adults in Nepal.

We transcribed all audio-recorded interviews verbatim in Nepali. To ensure accuracy, each transcript was cross-checked against the original audio recordings. We assigned each participant an alphanumeric code and removed all identifying information to maintain anonymity before beginning the analysis. We used framework analysis to examine the qualitative data. We started by familiarizing ourselves with the content, reading and re-reading the transcripts to identify emerging themes and key concepts. Next, we generated initial codes to capture ideas relevant to our research questions. We organized these codes according to the COM-B model domains and systematically linked the coded segments to the appropriate categories (Fig 1) [27]. Barriers and facilitators influencing hypertension management were classified across three domains: capability, opportunity, and motivation. Capability was further divided into physical capability (e.g., mobility limitations and fatigue affecting physical activity and healthcare visits) and psychological capability (e.g., forgetfulness, limited knowledge, and medication management skills). Opportunity was categorized into physical opportunity (e.g., proximity of health facilities, outreach programs, and time constraints) and social opportunity (e.g., family support, social isolation, dependence on caregivers, and support from FCHVs). Motivation was further categorized into reflective and automatic processes to guide thematic classification. Reflective motivation refers to conscious beliefs, evaluations, and decisions that influence behavior; therefore, codes related to lifestyle choices, perceived treatment benefits, medication hesitancy, and intentions to remain healthy for family members were grouped under reflective motivation. Automatic motivation refers to emotion-driven or instinctive influences on behavior; accordingly, codes reflecting emotional responses such as anger and fear of complications were categorized under automatic motivation, as these responses influenced adherence and blood pressure control without deliberate planning. This framework-guided approach informed the final thematic structure (Table 5).

This structured approach allowed us to maintain consistency throughout the analysis. Finally, we interpreted the data by identifying patterns, relationships, and insights within each COM-B domain [27]. We conducted the analysis using Taguette, an open-source software for qualitative data analysis.

## Result

The mean age of participants was 71.(6.7) years (Table 1). More than half (56.9%) were female and 68% of participants were married. Approximately 56% were able to read and write, and among them, 72.7% had received basic education. Over half belonged to the Janajati ethnic group, and most self-reported adhering to the Hindu religion. About 68% of participants lived with their son and daughter-in-law, and most (71%) resided in joint families.

**Table 1:** Socio-demographic characteristics of the older adults living with hypertensions (n = 197).

Characteristics	n	%
**Age Mean (SD)**	71 (6.7)	
**Sex**		
Female	112	56.9
Male	85	43.1
**Marital status**		
Married	134	68.1
Widowed	59	29.9
Divorced/ Separated	3	1.5
Never Married	1	0.5
**Education level**		
Able to read and write	110	55.9
Not Able to read and write	87	44.1
**Level of education n = 110**		
Basic education	80	72.7
Secondary education	20	18.1
Secondary and above	10	9.1
**Ethnicity**		
Janjati	103	52.2
Bhramin/chhetri	92	46.7
Dalit	2	1.02
**Religion**		
Hindu	179	90.9
Non Hindu	18	9.1
**Living arrangement**		
Living with son/ daughter in law	134	68.02
Couples only	39	19.8
Alone	9	4.5
Living with daughter/ son in law	7	6.9
Living with other relatives	4	2.0
**Type of family**		
Joint	140	71.1
Nuclear	46	23.3
Alone	9	4.5
Extended	2	1.0

n frequency % percentage.

The mean weight of participants was 59.6 (10.7) kg, and the mean height was 152.3(8.6 cm, Table 2). The average hypertension medication adherence score was 10.6 (2.2) indicating. The mean (S.D.) systolic and diastolic blood pressure measurements were 142.1 (18.3) mm Hg and 81.9 (10.4) mm Hg, respectively. More than half of the participants (56%) had at least one comorbidity.(Table 2)

**Table 2:** Physical measurements, blood pressure, and antihypertensive medication adherence among older adults living with hypertensions (n = 197).

Characteristics	n	%
**Weight (Kg) Mean (SD)**	59.6 (10.7)	
**Height (cm) Mean (SD)**	152.3 (8.6)	
**Body Mass Index (kg/m2) Mean (SD)**	25.7 (4.1)	
Underweight	6	(3.05)
Normal	88	(44.6)
Overweight	73	(37.1)
Obese	30	(15.2)
**Medication Adherence Mean (SD) Range (9–36)**	10.6 (2.2)	
**Systolic blood pressure In (mmHg) Mean (SD)**	142.1 (18.3)	
**Diastolic blood pressure In (mmHg) Mean (SD)**	81.9 (10.4)	
**Comorbidities**		
Present	110	(55.9)
Not present	87	(44.1)
**Type of comorbidities**		
Diabetes	62	(31.4)
Heart disease	23	(11.6)
COPD	19	(9.6)
Musculoskeletal problem	8	(4.1)
Others	34	(17.2)

N frequency % percentage.

**Fig 2:**
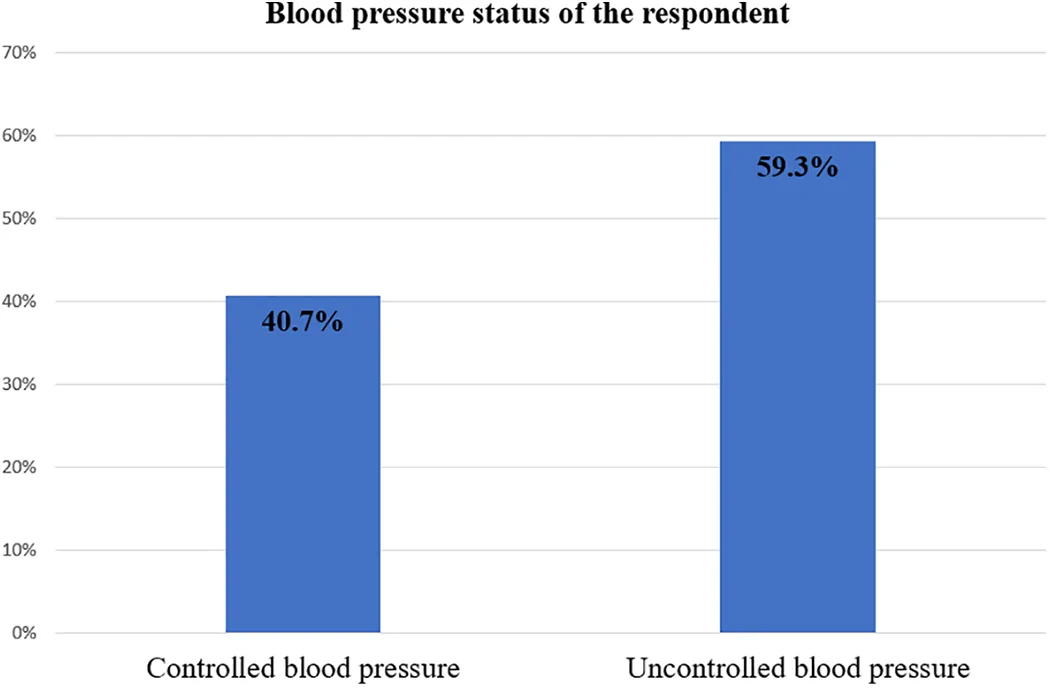
Controlled and Uncontrolled hypertension status of older adults living with hypertensions.

More than half of the participants (59.3%) had uncontrolled blood pressure (Fig 2).

No significant association was found between hypertension control status with age (p = 0.8), sex (p = 0.5), basic education (p = 0.1), secondary education and above (p = 0.3), marital status (p = 0.09), religion (p = 0.5), living arrangement (p = 0.5), presence of comorbidity (p = 0.4), medication adherence (p = 0.7) and BMI (p = 0.1) after adjusting for potential confounders (Table 3).

The mean age was 72.1 (4.7) years among older adults living with hypertension and 42.6 (13.1) years among caregivers (Table 4). More than half of the older adults living with hypertension were female (57.2%), while most caregivers were female (77.8%). Among older adults, 42.8% had secondary education or above, while this proportion was higher among caregivers (66.7%). More than half of the older adults (57.1%) belonged to the Brahmin/Chettri ethnic group, whereas all caregivers (100%) belonged to the Brahmin/Chettri ethnic group (Table 4).

**Table 3:** Logistic regression to determine factors associated with uncontrolled hypertension among older adults living with hypertensions (n = 197).

	Bivariable analysis			Multivariable analysis		
Characteristics	OR	95% CI	P value	OR	95% CI	P value
**Age**	1.0	0.9, 1.0	0.7	0.9	0.9,1.0	0.8
**Sex**						
Female	Ref			Ref		
Male	1.6	0.9, 2.8	0.1	1.3	0.5, 2.9	0.5
**Educational status**						
Not able to read and write	Ref			Ref		
Basic education	1.8	0.9, 3.4	0.05	1.8	0.8,4.03	0.1
Secondary education and above	1.6	0.6, 3.7	0.27	1.7	0.5, 5.5	0.3
**Marital status**						
Unmarried	Ref			Ref		
Married	0.7	0.3, 1.3	0.2	0.5	0.2,1.1	0.1
**Religion**						
Hindu	Ref			Ref		
Non-Hindu	1.08	0.4, 2.9	0.8	1.4	0.4, 4.0	0.5
**Living Arrangement**						
Living with family member	Ref			Ref		
Alone and couples only	0.7	0.3, 1.4	0.3	0.7	0.3,1.5	0.5
**Comorbidity**						
No	Ref			Ref		
Yes	1.2	0.7, 2.2	0.4	1.2	0.6,2.2	0.4
**Medication adherence**	0.9	0.8, 1.1	0.9	1.01	0.08,1.1	0.7
**BMI**						
Normal	Ref			Ref		
Non normal	0.6	0.3,1.1	0.1	0.6	0.3,1.2	0.1

Ref Reference group.

**Table 4:** Socio-demographic characteristics of participants of qualitative interviews.

Characteristics	Older adults living with hypertension (n = 7) n(%)	Caretaker (n = 18)
**Age (in years) Mean (SD)**	72.1 (4.7)	42.6 (13.1)
**Sex**		
Male	3 (42.8)	4 (22.2)
Female	4 (57.2)	14 (77.8)
**Educational status**		
Not able to read and write	1 (14.2)	2 (11.1)
Informal education	1 (14.2)	–
Basic education	2 (28.5)	4 (22.2)
Secondary education and above	3 (42.8)	12 (66.7)
**Ethnicity**		
Bhrahmin/Chettri	4 (57.1)	18 (100)
Janjati	3 (42.9)	–

**Table 5:** Presents the themes, sub themes identified and derived under COM-B model.

COM-B Model	Themes	Sub-themes	
		Barriers	Facilitators
**Capability**	Physical	• Physical limitation due to old age • Fatigue	• Physical activity to follow lifestyle choices
	Psychological	• Forgetfulness • Limited social interaction • Lack of awareness regarding hypertension management	• Use of pill box for reminder • Frequent monitoring of BP • Awareness and knowledge • Adhering to dietary recommendation
**Opportunity**	Physical	• Time constraint	• Nearby health facility • Community health programs through hospital • Support from FCHVs
	Social	• Lack of social support • Dependency on others	• Family support for navigating the health system
**Motivation**	Reflective	• Low interest in lifestyle change • Perceived stress causing hypertension • Low interest in treatment initiation • Fear of falling	• Perceived benefit • Desire to remain healthy for family member
	Automatic	• Anger	• Fear of complication

### Barrier

#### I. Capability.

a. **Physical capability**

**Physical limitation due to old age:** Many older adults said physical limitations that restricted their ability to engage in essential health behaviors, particularly physical activity and routine healthcare visits. Participants described experiencing body pain, pain in the legs, that hindered their mobility. Some shared that they had stopped walking or visiting health facilities because of these age-related issues.

*Maybe due to old age, my legs hurt whenever I go for a long walk. I just stay at home what else can I do* [p4, patient]

*When my legs hurt, I can’t go anywhere. I go out on a motorcycle, sit at a shop for a while, and then return home. It’s been about 1–2 years since this started happening.* [p4, patient]

**Fatigue:** In addition to joint pain, persistent fatigue was also identified as a significant barrier. Participants expressed that even performing basic household chores left them feeling exhausted, making it difficult to engage in any form of physical activity.

*I feel tired all the time. Doing small household chores makes me exhausted and tired. How can I be involved in physical activity in this way?* [p6, patient]

These limitations in physical capacity prevented individuals from following lifestyle recommendations such as daily exercise, which is essential for managing hypertension effectively.

b. Psychological capability

**Forgetfulness:** Forgetfulness and confusion were commonly said by older adults, especially concerning medication adherence. Participants admitted to frequently forgetting whether they had taken their medicines or not, sometimes leading to skipped doses or double doses.

*They often get confused if they have taken blood pressure medicine or not. The doctor has said if you feel that way, take the medicine again.* [p6, caretaker, FGD 1]

*I forget quite often to take and fill up the medicine. I start feeling dizzy and remember that I have to take the medicine.* [p1, patient]

Caregivers mentioned that even after being counseled, elderly patients struggled to remember their medication schedules

*Regarding medication schedules, the elderly might forget a bit. The doctors make them understand, but they still need to be reminded again.* [p5, caretaker, FGD 2]

**Limited social interaction:** Some participants highlighted the lack of meaningful social interaction and support within their community, further isolating them from discussions or shared learning about hypertension.

*Nobody talks with me here in the community, we do not interact with one another. How can I talk about blood pressure with anyone? All the neighbors are richer, and we are not, so how can I share my health problem with anyone? There is no one for any kind of support.* [p7, patient]

**Lack of information about hypertension management:** Very few participants expressed a general lack of understanding about hypertension, its causes, and its management. They reported not having access to credible sources of health information

*I don’t know anything about high blood pressure. How does it occur and all. Noone is there to tell me.* [p7, patient]

#### II. Opportunity.

a. **Physical opportunities**

**Time constraint:** Participants highlighted time constraints as a barrier to engaging in regular physical activity. Particularly among older adults with household responsibilities, daily chores overtook self-care, leaving little time for physical exercise, even when recommended by doctors. This limitation in available time reduced opportunities to follow recommended lifestyle changes crucial for hypertension control.

*I do not have much time to be involved in physical activities. I have to look after my house as well.* [p4, patient]

b. **Social influences**

**Lack of social support:** A lack of social support was a prominent barrier. In many cases, family members lived separately and were minimally involved in the older adults’ daily routines, including their diet and health monitoring. Caregivers acknowledged that they only provided assistance when a health issue became urgent.

*We are living separately for now, so we do not know much about their food habits. We take them to the hospital only when needed.* [p2, caretaker, FGD2]

Others shared that their social environment lacked people who could provide assistance or emotional support, particularly during emergencies. In households where elderly couples lived alone, there was a sense of vulnerability due to the absence of younger family members or supportive neighbors.

*My wife and I live alone. There is no one we can tell our problems to. We have neighbors and they are older than us. No young people are near so whom we can call for when we really need help.* [p3, patient]

**Dependency on family member:** This social isolation also affected their ability to seek and understand care. Many older adults were dependent on family members to accompany them to health facilities and communicate with healthcare providers. Without such support, they preferred not to visit the hospital at all. Such reliance on others not only limited their autonomy but also restricted their timely access to healthcare services, especially for routine check-ups and follow-up care.

*I only go to the hospital when a family member is with me. They talk to the doctor and understand what is wrong and what I need to do. I don’t understand it myself.* [p6, patient]

A few also described relying entirely on family members for their medication routines and hospital visits. Without their family’s assistance, they often skipped routine check-ups or left their treatment unmanaged.

*My sons and daughter remind me whether I have taken my medicine or not. I forget and that is why they keep track of my medication schedule.* [p4, patient]

*I only visit the hospital when any of the family members are there with me otherwise its better if I avoid visiting the hospital for my routine check up. I cannot do it on my own.* [p3, patient]

#### III. Motivation.

a. **Reflective motivation**

**1. Low interest in lifestyle change:** Many participants expressed low motivation to adopt recommended lifestyle changes such as reducing tobacco use, eating low-salt diets, or quitting smoking. Even when they were aware of the risks, personal preferences and habits outweighed health concerns.

*I know that smoking and using tobacco are harmful to my health but I do not want to give them up. Right now, it is the only thing that brings comfort and relief.* [p1, patient]

*I couldn’t quit smoking. I was restricted in many hospitals, and from a long time ago, I had other diseases. I had kidney stones, blood was not flowing in my bones, and then I got corona. I survived two or three serious diseases. After feeling better, I started craving certain foods, and I still feel like smoking.* [p7, patient]

Dietary behavior was similarly resistant to change. Participants often mentioned a preference for spicy, salty, and flavorful food, which made adherence to a heart-healthy diet difficult. Cultural norms around food habits also posed a challenge.

*I like food that is spicy, curry and all. It should be spicy. When there 2–3 items like these than I consume more salt. This has been our habit. [p3]*

*We tell her not to eat spicy or sour food, and she agrees. But when we say not to eat salty food, she complains that everything the daughter-in-law cooks is too bland. She says, “Just to eat food, I have to listen to all your rules? [p3, caretaker, FGD 1]*

**2. Perceived stress causing hypertension:** Some participants viewed stress as an unavoidable part of life, one that made it difficult to focus on health. Financial and family-related pressures often contributed to increased blood pressure, which they felt was out of their control.

*Due to family tension, I feel my blood pressure goes up. I can’t focus on my health when there are issues at home.* [p1]

*Due to stress in business where money does not come in and gets stuck in the market. my blood pressure increased. I cannot avoid working and cannot stop stressing it comes naturally.* [p5]

**3. Low motivation for treatment initiation:** Fear surrounding the lifelong commitment to medication also discouraged some from initiating treatment. The thought of dependency made them hesitant to begin taking antihypertensive drugs.

*The doctor told me to take medicine for high blood pressure, but I was scared I’d have to take it forever, so I thought it’s better not to start at all.* [p1]

*The doctor checked and said that I should start medication immediately. I was afraid that if I start taking it, I will have to take it forever, so I thought it might be better not to take it at all. [p1, patient, 77 years]*

Even among those who were adherent to medication, feelings of frustration arose when their blood pressure remained uncontrolled, which likely contributed to demotivation of continuing treatment.

*It’s my blood pressure. I get it checked every month, take my medicine regularly, yet my blood pressure always appears high. [p6]*

**4. Fear of falling**

*My mother has avoided going outside or doing any household chores ever since she fell last year. She is scared of falling again and that fear makes her inactive, which I know is not good for her health.* [FGD 1 caretaker]

b. **Automatic motivation**

**1. Anger:** Emotional responses such as anger were frequently reported to interfere with blood pressure control, despite regular medication use. Participants believed that their emotional temperament directly affected their blood pressure levels.

*I get angry really fast. I don’t know why. People often say this. This rises my bp even though I am on medication. [p1]*

Such automatic emotional responses are difficult to regulate and may contribute to poor hypertension control despite adherence to treatment.

### Facilitator

#### I. Capability.

a. **Physical capability**

**Physical activity to follow lifestyle choices:** Some participants actively engage in physical activities such as walking and stretching because they recognize the health benefits. Incorporating these activities into daily routines supports better hypertension management by improving physical health and mobility.

*I try to stay active every day because I know it helps me manage my health better. Even small exercises like walking or stretching have become part of my daily routine to keep me feeling good.* [p2, patient]

b. **Psychological capability**

**1. Use of pill box for reminder:** Participants use practical tools and strategies to enhance their ability to manage hypertension effectively. These include using pill boxes or color-coded bags to organize medications and prevent forgetting doses.

*I keep that medicine inside the box so as not to forget also I include dry fruits which are healthier.* [p1, patient]

*It’s easier when we keep the medicines in different colored plastic bags like green for morning and red for evening. That way, it’s easy to remember, and there hasn’t been any problem with this at my house.* [p5, caretaker, FGD 2]

**Frequent monitoring of bp:** Regular self-monitoring enabled participants to remain alert to any changes in their health status. This empowered them to take timely action and made them feel in control of their condition.

*I check my blood pressure frequently in weeks.* [p4, patient, IDI]

**Awareness and knowledge:** Increased awareness through media, counseling, and health check-ups enabled participants to better understand hypertension, recognize the importance of regular treatment, and adopt healthier behaviors.

*“Now, after going for a check-up, they know about hypertension. Nowadays, many people have a lot of information. If you look at the mobile, you’ll know; if you watch TV, you’ll know; if you go somewhere, you’ll know; nowadays, that’s how it is”* [p7, caretaker, FGD 1]

**Adhering to dietary recommendation:** Caretakers took the dietary advice given by health professionals and altered their family eating habits, such as reducing salt and oily food consumption, which shows an enhanced capability to make healthier choices.

*The doctor also said to avoid having too much salt in our food and pay attention to our diet. It has been like 5–6 years of not having oily meat, fish or too much salt.* [p9, caretaker]

#### II. Opportunity.

a. **Physical opportunities**

**Nearby health facility:** Proximity to health facilities played a significant role in facilitating hypertension management. Participants who lived near a basic health care center reported being able to access services quickly and conveniently. This ease of access encouraged timely visits for monitoring bp status.

*There is a health center just a few minutes from my home, so whenever I feel my blood pressure has risen, I can easily go there.* [p3, patient]

**Community health programs through hospital:** Periodic outreach activities and screening programs organized by hospitals created additional physical opportunities for patients to get diagnosed or monitor their blood pressure. These programs helped overcome accessibility barriers, especially for older adults or those who didn’t visit health facilities routinely.

*There are programs from hospitals where they screen high blood pressure. We take them there whenever there is a program.* [p6, caretaker, FGD1]

**Support from FCHVs:** FCHVs played a key role in extending the reach of hypertension care into communities. Their home visits and personal relationships with community members provided a bridge between formal health systems and individual households.

*Now, like our family member, FCHV sister is the one who comes and suggests us regarding bp status, programs that are happening in the community. They have been really helpful.* [p6, patient]

b. Social influences

**Family support for navigating the health system:** Family members provided logistical and emotional support that helped older adults manage their hypertension. They assisted with registering at health posts, accompanying patients to appointments, and managing long queues. This practical support significantly reduced the effort required for patients to access care.

*They told me to come in the morning, write my name, and it will be easier. The line is shorter in the morning, but later it gets crowded. So, either my son or daughter-in-law goes early in the morning, writes my name, I have a light meal, and then I go later.* [p2, patient]

Participants emphasized that family support was essential, especially for older adults with limited literacy or memory issues. Encouragement from family to take medications or attend appointments helped ensure adherence and continuity of care.

*Family support is extremely important for people like us, we need a lot of encouragement. When it comes to medication, sometimes we don’t understand, and forget as well. This is worse for those who are illiterate.* [p6, patient]

Such social influences created an enabling environment where patients were not managing their condition alone but were supported by a system of care within the household.

#### III. Motivation.

a. **Reflective motivation**

**Perceived benefit:** Many participants believed that proper medication and dietary control helped them maintain stable blood pressure and prevented further health deterioration. This understanding of positive outcomes from treatment reinforced their motivation to continue managing their condition.

*I take the medicine because it keeps my blood pressure under control and prevents other problems and does not harm my heart and kidneys.* [p2, patient]

**Desire to remain healthy for family member:** Participants expressed a sense of responsibility toward their families, especially their children and grandchildren. This emotional and social bond acted as a strong internal motivator to stay well, adopt healthy behaviors, and consistently take medications.

*I want to stay healthy, not just for myself, but for my children and grandchildren. If I fall sick, they will worry too. That is why I try to take my medicine, try to eat healthy and control my blood pressure.* [p5, patient]

b. Automatic motivation

**Fear of complication:** A key motivator for adhering to hypertension treatment was the fear of serious health consequences such as organ damage, disability, or death. Health provider’s warnings about the risks of uncontrolled blood pressure increased patient’s awareness of potential complications, prompting regular medicine use and lifestyle changes.

*They scared me by saying I must take the medicine, or something could happen, and it could affect any of my organs. They told me it could affect my eyes, heart, and other parts of my body, and that I must take blood pressure medicine that is why I take my medicine regularly* [p1, patient]

The integration of both quantitative and qualitative methods allowed for triangulation, providing a more holistic understanding of hypertension management among older adults. While the quantitative data showed the prevalence of uncontrolled hypertension, the qualitative data enriched these findings by exploring underlying behavioral and contextual factors. This mixed-methods approach enabled us to cross-validate and complement insights from both strands of data, offering a deeper interpretation of the results.

## Discussion

In this study among older hypertensive adults, more than half of participants had uncontrolled hypertension putting them at the risk of dementia. Likewise, more than half had at least one comorbidity. One-third were over-weight and one-sixth were classified as obese whereas only a small fraction were under-weight. No associations were found between hypertension control status and age, sex, education level, marital status, religion, living arrangement, presence of comorbidity, medication adherence and BMI. By using COM-B model, we explored the key barriers to hypertension management included limited physical and cognitive capability (fatigue, forgetfulness), lack of opportunity (limited social interaction, time constraints, poor access to information, and low family support), and reduced motivation (low interest in treatment or lifestyle changes, stress, and anger). Facilitators included improved capability through awareness, use of pill boxes, and regular BP monitoring. We also identified enhanced opportunity via nearby health facilities, community programs, and FCHV and family support as facilitators as well as strong motivation driven by fear of complications, perceived benefits, and the desire to stay healthy for the family.

In this study, more than half of older hypertensive participants (59%) had uncontrolled blood pressure. This result is similar to the findings from other studies, which have similarly high prevalence of uncontrolled hypertension. One study showed that 62% of individuals had uncontrolled blood pressure, while another reported a prevalence of 53% [28,29]. Contrary to the established evidence from South Asia, where systematic reviews and individual studies consistently report that medication adherence in older adults is generally suboptimal, ranging from 20% to 50%, better adherence is also linked to improved blood pressure control [30]. When integrating the qualitative findings, an important nuance emerges. No statistically significant associations were detected between hypertension control status and age, sex, education level, marital status, religion, living arrangement, comorbidity, medication adherence, or BMI. However, given the modest sample size and the number of predictors included in the multivariable model, the analysis may have been underpowered to detect smaller but clinically meaningful associations, and Type II error cannot be ruled out. Although the quantitative results showed high self-reported adherence scores and no statistical association with blood pressure control, qualitative interviews and group discussions consistently highlighted forgetfulness and confusion about medication timing as common challenges among older adults. Participants and caregivers described uncertainty about whether medicines had already been taken, sometimes leading to missed or repeated doses despite general intentions to follow prescriptions. This apparent discrepancy suggests that self-reported adherence measures may capture perceived or intended adherence rather than actual day-to-day medication-taking behavior. The qualitative strand therefore adds contextual depth by revealing unintentional non-adherence patterns that are not fully reflected in structured adherence scales. Together, the mixed-methods findings indicate that adherence among older adults may be overestimated in surveys while still being practically compromised by memory-related and cognitive barriers.

A study involving 114 older hypertensive patients found that about 48% had low adherence at baseline, but adherence improved significantly with interventions such as blood pressure self-monitoring assistance, which also supports better BP control [31]. Similarly, a study conducted in Thailand found a positive association between antihypertensive medication adherence and BP control status among elderly [32]. The lack of a significant association between uncontrolled blood pressure and medication adherence in this study may be partly explained by social desirability bias, as participants might have overstated their level of adherence during interviews. This overreporting could have masked the true relationship between adherence and blood pressure control.

Additionally, other unmeasured factors such as individual behaviors and lifestyle choices, health system characteristics, the environmental context in which older adults live, and relevant policies may have influenced blood pressure outcomes, regardless of medication adherence [33–35]. Furthermore, no association was found between blood pressure status and BMI. This contrasts with large-scale South Asian studies, which consistently report strong links between BMI and hypertension among older adults. A 2019 multi-country analysis showed each 5 kg/m^2^ BMI increase raised hypertension odds by 59–103% [36]. A 2024 study across five South Asian countries found that obesity (BMI ≥ 27.5 kg/m^2^) doubled the odds of hypertension [37]. However, findings vary regionally where a study in Sri Lanka showed significant BMI–blood pressure association, while some Indonesian studies did not [38,39].

Likewise, our study did not identify an association between the presence of comorbidity and blood pressure status. This contrasts with findings from previous studies that report an association between comorbidities and blood pressure control [40,41]. These inconsistencies both in relation to BMI and comorbidities may suggest that other contextual or behavioral determinants, such as access to healthcare, health literacy levels, or delays in seeking medical attention, are more influential in shaping blood pressure outcomes in this population. These inconsistencies in the studies point to the importance of exploring behavioral and contextual factors, an area where the qualitative findings provided critical insights.

### Capability

Under capability domain, several barriers, including old age-related physical limitations and fatigue, restricted participants’ ability to engage in physical activity. This is consistent with systematic reviews and a national representative study, which report that mobility issues, chronic pain, and fatigue reduced physical activity among older adults [42,43]. Cognitive challenges, such as forgetfulness were common and exacerbated by confusion from polypharmacy, complicating medication adherence. This is similar to the systematic review including multiple international studies that concluded that memory function is the strongest predictor of medication adherence in older adults with hypertension [44]. Likewise, in a hospital based study conducted in North India showed that polypharmacy and cognitive function as a major factors for medication adherence [45]. Cultural practices such as fasting further deterred the use of prescribed medications. This aligns with a study conducted in Indonesia which shows that fasting during Ramadan are associated with lower medication adherence among patients with chronic conditions including hypertension. Limited mealtimes and fasting schedules can disrupt the timing and regularity of medication intake, making it difficult for older adults to adhere to prescribed antihypertensive regimens [46]. A lack of social interaction further reduced opportunities for health related discussions and reminders. This is similar to the systematic review that shows social support is associated with self-management ability among hypertensive older age group [47]. Additionally, a lack of awareness regarding hypertension and its management limited individuals’ ability to make informed decision about their care, similar to the study from Kerala, India where knowledge and awareness about the necessity for health check-ups and management of hypertension emerged as a theme [48]. Despite these barriers, several facilitators within the capability domain supported better management of hypertension. Engagement in physical activity emerged as a positive behavior that supported overall health and lifestyle modifications. Similar findings are documented in a systematic review conducted in South Asia where older adults who are engaged in regular walking and light exercise reported improved physical well-being [49]. The use of pill boxes served as an effective memory aid, helping individuals adhere to their medication schedules. This has been supported by qualitative exploration among older adults with HIV and Type 2 Diabetes Mellitus who often use pillboxes alongside reminder tools such as alarms reflecting various approach to medication adherence [50]. Themes such as routine monitoring, and following dietary recommendations were factors facilitating better management of hypertension similar to other studies conducted in South East Asia [51].

### Opportunities

Under the opportunity domain of the COM-B model, key barriers to hypertension management among older adults in Nepal include lack of time, inadequate family support, and dependency on others. Many participants, despite their age, remained busy with household or agricultural duties, leaving little time for self-care. This finding is similar to a study from India where older adults prioritized daily chores over managing chronic conditions [52]. Limited family involvement also hindered care where participants often lacked reminders for medication, accompaniment to health facilities and emotional support. This was similar to the study conducted in Bangladesh, Indonesia and South Korea where family members often provided practical emotional and physical support including medication and accompaniment [53]. Additionally, many older adults depend on others for transportation or financial help, which delays or prevents access to services. This aligns with findings from Ghana, where reliance on caregivers was identified as a barrier to timely hypertension care [54]. Alongside these challenges, certain factors within the same domain enabled more effective hypertensive management. Easy access to nearby health facilities enabled timely consultations and follow-ups, a factor similarly noted in a study from rural India [55]. Likewise community support, such as FCHVs and screening programs, played an important role for hypertension for screening and providing information regarding hypertension and its management. Various studies find the effectiveness of FCHVs in hypertension management [56,57]. Family support was another major facilitator for managing and seeking routine hypertension care specially for navigating health care centers.

### Motivation

Barriers such as low interest in lifestyle change, low interest in treatment initiation, and anger emerged as major barriers for maintaining the blood pressure control. Comparable findings were reported in a study from Ghana, which highlighted poor adherence to self-management practices and irregular follow-ups as major challenges issues closely tied to an individual’s motivation and willingness to engage in long-term care [58]. Similarly, the American Heart Association emphasizes that psychosocial stressors like anxiety and anger can significantly reduce patients’ motivation and adherence to prescribed regimens, underscoring the complex interplay between emotional health and chronic disease management [3]. Various facilitators identified include perceived benefit, desire to remain healthy for family members, and fear of complications. Similarly, a qualitative study conducted in rural India found that older adults were more likely to adhere to hypertension treatment when they understood the benefits and wanted to avoid being a burden to their families [59]. Likewise, research from Bangladesh highlighted fear of stroke or heart attack as a critical motivator for continued medication use and lifestyle modification among hypertensive patients [60].

### Strengths and limitations

To the best of our knowledge, this is among the few of the comprehensive studies in Nepal to assess blood pressure control status while simultaneously exploring the barriers and facilitators to managing hypertension among older adults living with hypertension in Nepal. A strength of this study is our use of rigorous methodology. We conducted an in-depth analysis that integrated various factors including socio-demographic characteristics, medication adherence, BMI, and comorbidities to assess their associations with uncontrolled blood pressure status. The multivariable regression approach allowed us to identify potential independent associations after adjusting for confounders By applying the COM-B model, we explored a range of barriers and facilitators influencing how older adults manage hypertension, shedding light on how individuals interact with health behaviors. Our iterative approach and achievement of code saturation enabled us to capture diverse perspectives and experiences. Moreover, by using both IDIs and FGDs, we incorporated insights from older adults living with hypertension, caregivers, and healthcare providers, enriching the overall findings. Overall, our research emphasizes the importance of holistic, context-sensitive strategies that consider the unique behavioral and environmental realities of older adults in LMICs, providing a framework that other countries facing similar challenges can adapt to enhance hypertension management and reduce related health risks.

Nevertheless, this study has several limitations. First, because the research was conducted in a single geographic area and included only individuals enrolled in the Healthy City Initiative, the generalizability of the findings is limited. In addition, the modest quantitative sample size and the number of predictors included in the multivariable model mean the analysis may have been underpowered to detect smaller but clinically meaningful associations, and a Type II error cannot be ruled out. The study also relied on self-reported data, which may introduce social desirability bias, particularly in measures such as medication adherence and perceived challenges. Similarly, as with many qualitative studies, participants’ narratives may have been influenced by a desire to present themselves favorably or by discomfort in disclosing personal experiences, potentially resulting in under-reporting or over-reporting.

## Conclusion

This study among older adults with hypertension notes that over half of the participants had uncontrolled blood pressure. No associations were found between hypertension control status and age, sex, education level, marital status, religion, living arrangement, presence of comorbidity, medication adherence and BMI.

However, when explored through the lens of the COM-B model, a range of behavioral determinants emerged as critical to understanding hypertension management including physical and cognitive capability, lack of opportunity, and reduced motivation. On the other hand, several facilitators played a positive role in promoting blood pressure control such improved capability through awareness, use of pill boxes, and regular BP monitoring; enhanced opportunity via nearby health facilities, community programs, and FCHV and family support; and strong motivation driven by fear of complications, perceived benefits, and the desire to stay healthy for family.

The findings underscore the importance of adopting a holistic, behavior-centered approach to hypertension care in older populations. While pharmacological treatment remains important, emphasizing non-pharmacological interventions such as promoting physical activity, a balanced diet, stress reduction, and social support can significantly improve health outcomes. These strategies align closely with behavior-centered approaches and highlight the value of integrating behavioral science frameworks like COM-B into chronic disease management. In this context, the present study offers an important reference point for researchers, health practitioners, and policymakers aiming to develop contextually relevant, patient-centered interventions. Such integration can foster more sustainable, effective, and equitable health outcomes for older adults in Nepal and other similar low-resource settings, eventually reducing the scale of dementia prevalence.

## Supporting information

S1 Text Interview guide. (DOCX)

S1 Data Dataset. (XLSX)

## Abbreviations

BMI: Body Mass Index
COM-B: Capability, Opportunity, Motivation Behavior model
IDIs: In depth Interviews
FGDs: Focus Group Discussions
FCHVs: Female Community Health Volunteers
RA: Research Assistant
RUDAS: Rowland Universal Dementia Assessment Scale
BP: Blood Pressure
