# Supplementary material for: Barriers and facilitators of hypertension control among older adults living with hypertension: A mixed-methods study
Source: PLOS Glob Public Health. 2026 Aug 20;6(8):e0007015. doi: 10.1371/journal.pgph.0007015 (PMC13492771; doi:10.1371/journal.pgph.0007015)
Supplement: Supplementary file 1 [file pgph.0007015.s001.docx]

Top of Form

Reespondent id number*

**Socio-Demographic information**

1. Age (in completed years)*
2. Sex of the participant*

- Male
- Female
- Other ________

3. Marital status of the participant*

- Never married
- Married
- Divorced/SeparatedWidowed

4. Educational status of the participant*

- Able to read and write
- Not able to read and write

If able to read and write,

- Basic education
- Secondary education
- Secondary and above education

5. Ethnicity of the participant*

- Bhrahmin/Chhetri
- Dalit
- Janjati
- Madhesi
- Muslim
- Other

6. Religion of the participant*

- Hindu
- Buddhist
- Muslim
- Kirat
- Christian
- Other

7. Type of family*

- Nuclear
- Joint
- Extended
- Other

**General information of participant**

8. Whom do you live with? (Living arrangement)*

- Living with son /daughter-in-law
- Living with daughter/ son in-law
- Couples onlyLiving with other relatives
- Alone
- Other

9. Are you suffering from any other chronic health problem in addition to hypertension?*

- Yes
- No

If yes, its it {multiple choice}

- Diabetes
- Heart Disease
- Chronic Obstructive Pulmonary Disease
- Musculoskeletal problem
- Others _________

Now, measure the information of the participants with devices provided

10. Height of the respondent* _____ in cm
11. Weight of the respondent* ____ in kg
12. Systolic blood pressure*____ in mmHg
13. Diastolic blood pressure* ____ in mmHg

**Hill-Bone Medication Adherence Scale (HB-MAS)**

Direction: The researcher is requested to tick the appropriate response on the Likert scale for each question based on the participant's response.Hill-Bone Medication Adherence Scale (HB-MAS)

**None of the time (4)Some of the time (3)Most of the time (2)All of the time (1)**

How often do you forget to take your high blood pressure medicine?*

How often do you decide NOT to take your high blood pressure medicine? question*

How often do you forget to get prescriptions filled?*

How often do you run out of high blood pressure pills?*

How often do you skip your high blood pressure medicine before you go to the doctor?*

How often do you miss taking your high blood pressure pills when you feel better?*

How often do you miss taking your high blood pressure pills when you feel sick?*

How often do you take someone else's high blood pressure pills?*

How often do you miss taking your high blood pressure pills when you are careless?*

Bottom of Form

‘

**Qualitative**

## Barriers and Facilitators of Hypertension Control among Older Adults Living with Hypertension

### Background Information

- Age (in years):
- Sex: Male, Female
- Educational status: Not able read and write/ Informal education/ Basic education/ Secondary education and above
- Ethnicity: Bhrahmin/Chhetri, Dalit, Janjati, Madhesi, Muslim, Othe

## A. Individual Level

1. Can you tell me the how you first came to know that you had high blood pressure?
2. What does living with high blood pressure mean to you in your daily life?
3. Please describe your experiences in trying to control your blood pressure?
4. What makes it easier for you to manage your blood pressure?
5. What makes it difficult for you to manage your blood pressure or seek care?

*daily activities, responsibilities, financial or physical conditions , follow up*

## B. Interpersonal Level

1. Who are the people involved in supporting you with your blood pressure care?

*In what ways do they support you?*

2. How does their support influence your ability to manage your condition?
3. Have there been times when you did not receive the support you needed? Can you describe that experience?
4. How does communication within your family affect your blood pressure management?

## C. Community Level

1. How is high blood pressure generally understood in your community?
2. How do people in your community usually respond when they are diagnosed with high blood pressure?
3. What influences people’s decisions to seek or not seek care for high blood pressure?
4. What community factors make it easier to access care?
5. What community factors make it difficult to access care?
6. Are there any community groups, services, or individuals that help people manage chronic conditions? Please describe.

## D. Organizational Level (Health Facility Level)

1. Can you describe your experiences when visiting health facilities for blood pressure treatment?
2. What aspects of the health facility make it easier for you to continue treatment?
3. What challenges have you faced while receiving services?
4. How would you describe your interactions with health workers?
5. How do service availability, waiting time, or cost affect your follow-up and treatment?

## E. Policy / Structural Level

1. What kind of support have you received from government programs related to hypertension care?
2. How have these programs helped or not helped you?
3. What challenges do you face in accessing government-supported services?
4. What changes in policies or programs would make it easier for you to control your blood pressure?
5. Is there anything else about managing high blood pressure that we have not discussed but you think is important?

# FGD Guide – Caregivers

### Background Information

### Background Information

- Age (in years):
- Sex: Male, Female
- Educational status: Not able read and write/ Informal education/ Basic education/ Secondary education and above
- Ethnicity: Bhrahmin/Chhetri, Dalit, Janjati, Madhesi, Muslim, Othe

## A. Individual Level

1. Can you describe your role in helping the older family member manage their high blood pressure?
2. How do you support them in their daily care and treatment?
3. How confident do you feel in managing their appointments, medications, and emergencies?
4. What helps you feel confident in this role?
5. What makes it difficult for you to provide care?
6. How does your own health or daily responsibilities affect your caregiving role?

## B. Interpersonal Level

1. How would you describe communication between you and the older family member regarding their health?
2. How does this communication influence blood pressure management?
3. What interpersonal challenges do you face while supporting their treatment and follow-up?
4. What kind of family support helps in managing their condition?

## C. Community Level

1. What challenges do you and the patient face when accessing local health services?
2. How does the community support or hinder hypertension management?
3. Are there any community-based programs that support older adults with hypertension? Please describe your experience with them.

## D. Organizational Level

1. What has been your experience when accompanying the patient to healthcare facilities?
2. What makes accessing services easier?
3. What makes accessing services difficult?
4. How would you describe your interactions with healthcare providers?
5. How do availability of medicines, diagnostic services, waiting times, or costs affect care?

## E. Policy Level

1. What has been your experience with accessing services through national health insurance or government programs?
2. What challenges have you faced in using these services?
3. In your opinion, how well do current policies address the needs of older adults with hypertension?
4. What support should the government provide to patients and caregivers?
5. Is there anything else you would like to share?
