# Supplementary material for: Barriers and facilitators of hypertension control among older adults living with hypertension: A mixed-methods study
Source: PLOS Glob Public Health. 2026 Aug 20;6(8):e0007015. doi: 10.1371/journal.pgph.0007015 (PMC13492771; doi:10.1371/journal.pgph.0007015)
Supplement: Supplementary file 2 [file pgph.0007015.s003.docx]

Nov 11, 2025

**Barriers and Enablers of Hypertension Control in Nepalese Older Adults: Implications for Dementia Prevention**

**Reviewer Comments**

**For author and editor**

Thank you for requesting me to review this manuscript. Barriers and Enablers of Hypertension Control in Nepalese Older Adults: Implications for Dementia Prevention is an important subject.

To make the manuscript more scientifically sound, authors should pay attention to the following areas:

**Overall comments**

- **Title**: Title does not represent your finding. Quantitative part is neglected from title. As title mentioned “Implication for dementia prevention” but you have excluded reported dementia patient based on the Rowland Universal Dementia Assessment Scale (RUDAS). If you exclude the participant who reported that they have dementia, how you ensure that this article justify the title. It does not have meaning.
- **Abstract**: In methods, limited information included about sampling process, study area, study duration, tool and technique used in the study. It would be useful if authors provide information in methods portion. This will help readers to have a better picture of the original study where data was drawn and used in this study.
- **Methods**: Authors didn’t mentioned sample size estimation process (how you calculate the sample size from Healthy City 109 Initiative Dataset). Study duration is missing. Authors should mention the clear process how you calculate the sample size.
- **Results:**
- Table 1, 2: Please write frequency and percentage separately.
- Please write table description above the table.
- Table 3: The way of presenting the table is not easy to read. It is better to write the full form of ref at the footnote of table.
- Table 4: Title is confusing. You have already mentioned socio-demographic information in Table 1. Don’t write patient. You can write participants or respondents.
- Please write two digit after decimal.

**Reviewer impression**: From title to the conclusion quantitative finding was neglected.

Thanks
