# Supplementary material for: Barriers and facilitators of hypertension control among older adults living with hypertension: A mixed-methods study
Source: PLOS Glob Public Health. 2026 Aug 20;6(8):e0007015. doi: 10.1371/journal.pgph.0007015 (PMC13492771; doi:10.1371/journal.pgph.0007015)
Supplement: Supplementary file 3 [file pgph.0007015.s005.docx]

Dear Editor and Reviewers,

Thank you very much for the careful review of our manuscript and for giving us the opportunity to submit a revised version to *PLOS Global Public Health*. We sincerely appreciate the constructive and detailed feedback provided. We have carefully addressed all comments and suggestions and have revised the manuscript to improve clarity, framing, methodological transparency, and interpretation of the findings. Below, we provide a detailed, point-by-point response to each comment and indicate where corresponding revisions have been made in the manuscript.

Thank you again for your valuable feedback and for the opportunity to improve our work.

| Reviewer’s comments | Author’s response to the comments |
| --- | --- |
| Overall comments: Overall comment The second portion of the title- “implication for dementia prevention” is not explored much in the study itself, except in the literature review section, where readers are provided with the information that high BP is a risk factor for dementia. I don’t know if that alone warrants the inclusion of the phrase in the title, when this study didn’t investigate the association or implication of such at all. | We thank the reviewer for this important observation. We agree that the study did not directly assess the association between blood pressure status and dementia or determined dementia prevention outcomes. In response to this comment, we have removed the phrase “implication for dementia prevention” from the title to ensure that it accurately reflects the scope and findings of the study. |
| Title: Title does not represent your finding. Quantitative part is neglected from title. As title mentioned “Implication for dementia prevention” but you have excluded reported dementia patient based on the Rowland Universal Dementia Assessment Scale (RUDAS). If you exclude the participant who reported that they have dementia, how you ensure that this article justify the title. It does not have meaning. | Regarding the use of the RUDAS, this tool was applied solely as a screening criterion to exclude participants with cognitive impairment. Participants with cognitive impairment were excluded to ensure the reliability and accuracy of self-reported information, as cognitive impairment may affect recall and response validity. The study did not aim to assess dementia prevalence or its association with hypertension. We have revised the title to better represent both the quantitative and qualitative components of the study and to accurately reflect the scope of the research. |
| Abstract: In methods, limited information included about sampling process, study area, study duration, tool and technique used in the study. It would be useful if authors provide information in methods portion. This will help readers to have a better picture of the original study where data was drawn and used in this study. | Thank you for the suggestion. We have now modified method section as follows: We conducted a parallel mixed-methods study in Dhulikhel municipality, Nepal, between September 2024 and March 2025. The quantitative component assessed blood pressure control among 197 older adults (aged ≥60 years) living with hypertension. We measured blood pressure (mmHg) and body mass index (kg/m²) and assessed medication adherence using the Hill-Bone Medication Adherence Scale. We summarized categorical variables using frequencies and percentages and continuous variables using means and standard deviations. We applied multivariable logistic regression to determine factors associated with blood pressure control. For the qualitative component, we explored barriers and facilitators to hypertension management among older adults using the Capability, Opportunity, and Motivation–Behavior (COM-B) model. Trained researchers conducted seven in-depth interviews with older adults living with hypertension and two focus group discussions with caregivers. We held all interviews and FGDs in private settings, using a semi-structured guide in the Nepali language. We audio-recorded each session, transcribed the recordings verbatim, and applied both inductive and deductive coding. Finally, we analyzed the data using a framework approach supported by Taguette software. |
| Methods: Authors didn’t mentioned sample size estimation process (how you calculate the sample size from Healthy City 109 Initiative Dataset). Study duration is missing. Authors should mention the clear process how you calculate the sample size. | Thank you so much for pointing that out. We have added sample size calculation in participant recruitment and sampling strategy as follows “The sample size was calculated using the single population proportion formula. We assumed a 95% confidence level, a margin of error of 7%, and an expected prevalence of hypertension of 41.7% among older adults in Nepal. The minimum required sample size was calculated to be 191. After adding a 10% allowance for non-response, the target sample size was 210. However, we were able to successfully reach and enroll 197 eligible participants in the final survey” |
| Results:  Table 1, 2: Please write frequency and percentage separately | Thank you so much for your suggestion. We have written frequency and percentage in different columns. |
| Please write table description above the table. | Thank you so much for your suggestions. We have moved the table description above the table. |
| Table 3: The way of presenting the table is not easy to read. It is better to write the full form of ref at the footnote of table. | Thank you so much for your comment. We have mentioned the full form of Ref in the footnote. |
| Table 4: Title is confusing. You have already mentioned socio-demographic information in Table | Thank you so much for your comment. Table 4 is socio demographic description of qualitative study participants. |
| 1. Don’t write patient. You can write participants or respondents. | Thank you for your suggestion. We have now changed patient with people living with hypertension |
| Please write two digit after decimal. | Thank you for the suggestion. However, we have kept one decimal place because additional decimal precision does not change the interpretation or statistical conclusions of the results. We therefore chose the simpler format for clearer presentation. |
| Reviewer impression: From title to the conclusion quantitative finding was neglected. | Thank you for the helpful comment. In response, we have revised the manuscript to ensure that the quantitative findings are consistently emphasized from the title through the conclusion. We added clearer details in the Methods section regarding the quantitative component and analysis, and expanded the Discussion to interpret and contextualize the key quantitative results |
| Reviewer 1 | |
| Overall comment The second portion of the title- “implication for dementia prevention” is not explored much in the study itself, except in the literature review section, where readers are provided with the information that high BP is a risk factor for dementia. I don’t know if that alone warrants the inclusion of the phrase in the title, when this study didn’t investigate the association or implication of such at all. | Thank you for this valuable comment. We agree that the phrase “implication for dementia prevention” was not sufficiently examined within the scope of our study and therefore may overstate the study focus. In response, we have revised the title to better reflect the actual objectives and findings of the research. The revised title is: “Barriers and Facilitators of Hypertension Control among Older Adults Living with Hypertension: A Mixed-Methods Study.” |
| Abstract conclusion- the ‘how’ of minimizing dementia risk is missing. Consider adding BP control as a pathway for minimizing dementia. For example, you could say- “Incorporating behavioral frameworks…settings like Nepal to improve BP control and therefore minimize the risk of dementia in this population.” | Thank you for the suggestion. We have updated the abstract conclusion to clearly state that the recommended strategies aim to improve BP control in low-resource settings like Nepal. Because dementia outcomes were not directly investigated, we removed dementia prevention from the title to better align with the study scope. |
| - Line 108-110- Please add more context about the “Healthy City Initiative dataset.” Does it include all households of the municipality? Or is it a list of people diagnosed with certain conditions residing in the city? Was the random selection done from the entire list or from a subset of the list created by filtering for selecting age and diagnosis? Who maintains the dataset, and how did the researchers access it? This information will help the readers to understand sampling bias (if any) and understand the generalization of the findings. | Thank you for your suggestion, we have added this in the participant recruitment and sampling strategy “We used the Healthy City Initiative dataset of Dhulikhel Municipality. This dataset, jointly developed and maintained by Dhulikhel Hospital and Dhulikhel Municipality, contains household-level information on socioeconomic characteristics, health behaviors, morbidity, and mental health of municipal residents to support early identification and management of non-communicable diseases. Permission to access and use the dataset was obtained from the Principal Investigator of the Healthy City Initiative and Dhulikhel Municipality. From this dataset, records were first filtered to identify older adults meeting the study eligibility criteria, including age and hypertension status. Eligible participants were then randomly selected from this filtered subset using Stata software.” |
| - Line 130-133: It is unusual (and thus less convincing) that the researchers achieved ‘saturation’ with only 7 participants (for in-depth interviews) and two focus group discussions, and stopped their recruitment. Were additional criteria used to determine the sample size? | Thank you for your comment. We acknowledge that the sample size for qualitative studies can appear small. In our study, we conducted 7 IDIs and 2 FGDs comprising 18 caregivers. Recruitment was guided by the principle of data saturation, a widely accepted approach in qualitative research. Saturation was systematically monitored throughout data collection, and recruitment was stopped once no new codes, themes, or insights emerged from additional interviews.  We also ensured maximum variation in our participants, including differences in age, gender, relationship to the patient, and experience with care, to capture a broad range of perspectives. Several methodological studies suggest that for focused qualitative topics, saturation can often be achieved with a relatively small number of interviews or 2–3 FGDs, particularly when the research question is narrow and participants share similar experiences (Guest et al., 2006; Hennink et al., 2017).  Therefore, we believe that our sample size was adequate to comprehensively address the study objectives while maintaining methodological rigor. |
| - Line 142- Clarify the difference between a ‘joint’ family and an ‘extended’ family. | A joint family refers to a household in which parents live with their children and grandchildren. In contrast, an extended family includes parents, their siblings, cousins, and their respective children residing within the same household. |
| - Line 203- The sentence is incomplete. Indicating what? | Thank you so much for your comment. We have removed the word indicating from the paragraph. |
| - Table 3- Two variables- type of family, and living arrangement, likely have high intercorrelation. Multicollinearity might be a concern for the multivariate regression analysis. Thus, I suggest selecting one of those variables instead of including both in the model. | Thank you for your comment. We appreciate your concern regarding potential multicollinearity. To clarify, the type of family variable was not included in the multivariable regression analysis. It was only used in the descriptive analysis to summarize participant characteristics. The regression model only included living arrangement. |
| - Line 182, figure 1, and Table 5- Need additional explanation on themes. Physical and psychological are straightforward, but what do ‘reflective’ and ‘automatic’ motivation mean? This will help the readers understand why ‘anger’ is ‘automatic’ motivation and others ‘reflective.’ | Thank you for this helpful suggestion. We have now added a clearer explanation of reflective and automatic motivation based on the COM-B framework in the Data Analysis section as this “We organized these codes according to the COM-B model domains and systematically linked the coded segments to the appropriate categories (Figure 1).[(27)](https://paperpile.com/c/XvZBvZ/4LHqY) Barriers and facilitators influencing hypertension management were classified across three domains: capability, opportunity, and motivation. Capability was further divided into physical capability (e.g., mobility limitations and fatigue affecting physical activity and healthcare visits) and psychological capability (e.g., forgetfulness, limited knowledge, and medication management skills). Opportunity was categorized into physical opportunity (e.g., proximity of health facilities, outreach programs, and time constraints) and social opportunity (e.g., family support, social isolation, dependence on caregivers, and support from FCHVs). Motivation was further categorized into reflective and automatic processes to guide thematic classification. Reflective motivation refers to conscious beliefs, evaluations, and decisions that influence behavior; therefore, codes related to lifestyle choices, perceived treatment benefits, medication hesitancy, and intentions to remain healthy for family members were grouped under reflective motivation. Automatic motivation refers to emotion-driven or instinctive influences on behavior; accordingly, codes reflecting emotional responses such as anger and fear of complications were categorized under automatic motivation, as these responses influenced adherence and blood pressure control without deliberate planning. This framework-guided approach informed the final thematic structure (Table 5).” |
| - Line 389. Instead of participants ‘noted’, consider writing participants ‘believed’ that- because they believe that their temperament is causing high BP. | This has been changed in the manuscript as per your comment |
| - Line 545-546, the systematic review that was cited after the sentence –“however, no significant association was found between uncontrolled blood pressure and medication adherence in this study” studied factors influencing adherence, not the association between BP and adherence. So this citation is misplaced. | Thank you for your suggestion, the citation has been moved to the correct place. |
| - Line 552- add citation for social desirability bias as one of the explanations for non-association. | Citation https://pmc.ncbi.nlm.nih.gov/articles/PMC6062205/?utm_source=chatgpt.com |
| - In lines 566 to 569, the authors write- “these inconsistencies... may suggest... are more influential...” I have a problem with the “are more influential" claim, which didn’t come from this study. Nor do the authors provide any citations for this claim. Please add citations. If not, and if it is the author's speculation only, then consider saying- “could be additional factors that influence BP outcome.” | We thank the reviewer for pointing this out. We agree with the phrase, and modify it as follows “These inconsistencies, both in relation to BMI and comorbidities, could suggest that other contextual or behavioral factors such as access to healthcare, health literacy, or delays in seeking medical attention might also influence blood pressure outcomes in this population. These possibilities highlight the importance of exploring behavioral and contextual factors, an area where the qualitative findings provided critical insights”. |
| - Line 580-584, the discussion about the influence of ‘fasting’ practice seems out of place. The authors didn’t explore fasting as a probable cause for nonadherence, nor did their qualitative findings reveal such. | “I do not take medicines during Teej fasting. I don’t feel comfortable taking them because I believe it may break my fast.” |
| - Lines 639 to 640. The authors say, “The analysis identified independent associations even after adjusting for potential confounders.” But the study didn’t find any independent (significant) association; the sentence sounds misleading. If they are talking about the benefit of using multivariate regression, perhaps they should write – “the multivariate regression approach allowed us to identify potential independent associations after adjusting for confounders.” | Thank you so much for your valuable comment. We have rewritten as per your suggestion. |
| Reviewer #2: |  |
| This manuscript addresses an important public health issue: hypertension management among older adults in a low-resource setting, and the use of the COM-B framework is a clear strength. However, several methodological and interpretive issues need revision. | Thank you for your acknowledgement and comments. We have revised the paper as per your comments. |
| 1. Quantitative findings & validity Table 3 shows no significant associations between uncontrolled hypertension and age, sex, education, BMI, comorbidity or adherence. Given existing evidence, the lack of association with BMI and adherence is implausible and likely reflects limited statistical power and/or measurement issues.With n = 197, the multivariable model is probably underpowered. Results should be framed as “no associations detected,” with a brief post-hoc comment on power and Type II error. | Thank you for your suggestion, we have now added this in the discussion section “ No statistically significant associations were detected between hypertension control status and age, sex, education level, marital status, religion, living arrangement, comorbidity, medication adherence, or BMI. However, given the modest sample size and the number of predictors included in the multivariable model, the analysis may have been underpowered to detect smaller but clinically meaningful associations, and Type II error cannot be ruled out. |
| The Hill-Bone scores indicate near-perfect self-reported adherence despite ~60% uncontrolled BP, suggesting strong ceiling and social desirability effects.  This limitation and its implications for the null adherence–BP finding should be discussed more explicitly. | We thank the reviewer for this comment. We have now elaborated the Limitation section in the Discussion section to acknowledge the effect and social desirability bias associated with the Hill-Bone Adherence Scale. We highlight that the because of high adherence scores likely reduced variability, thereby limiting the ability to detect an association between adherence and blood pressure control. |
| 2. Qualitative sampling & “enablers” All qualitative interviews were conducted with older adults with uncontrolled BP. This constrains the ability to identify true “enablers” of good control, as any reported facilitators have not led to control for these participants. | We thank the reviewer for this important comment. We have clarified in the manuscript that caregivers of individuals with both controlled and uncontrolled hypertension were interviewed. However, we now explicitly acknowledge that the qualitative findings predominantly reflect barriers related to uncontrolled hypertension and that the absence of a positive deviance from patient perspective analytical approach is a key limitation. |
| The manuscript should clearly acknowledge that the qualitative findings mainly reflect barriers among those with uncontrolled hypertension and that the absence of “positive deviants” is a key limitation. | This study explores barriers to hypertension management among older adults with uncontrolled blood pressure. As participants with well-controlled hypertension were not included, the findings mainly reflect challenges rather than successful strategies for effective hypertension management. The absence of positive deviants limits the ability to identify facilitators and adaptive practices that could inform intervention design |
| 3. “Dementia prevention” framing No dementia or cognitive outcomes are analyzed, and dementia-related pathways are not examined beyond the background. The emphasis on “dementia prevention” in the title and conclusions is therefore overstated. I recommend removing “dementia prevention” from the title and tempering related claims in the abstract and Discussion. | Thank you so much for your suggestion. We have removed dementia prevention from the manuscript. |
| 4. Mixed-methods integration The study is presented as mixed-methods, but the two strands are not well integrated. Suggestions: Add a clear paragraph in the Discussion that brings quantitative and qualitative results together, especially the conflicting findings, For example, qualitative data highlight “forgetfulness” as a key barrier, yet the quantitative data show very high self-reported adherence and no link between adherence and BP control | Thank you so much for your suggestion. In the second paragraph of discussion we have added this “ When integrating the qualitative findings, an important nuance emerges. Although the quantitative results showed high self-reported adherence scores and no statistical association with blood pressure control, qualitative interviews and group discussions consistently highlighted forgetfulness and confusion about medication timing as common challenges among older adults. Participants and caregivers described uncertainty about whether medicines had already been taken, sometimes leading to missed or repeated doses despite general intentions to follow prescriptions. This apparent discrepancy suggests that self-reported adherence measures may capture perceived or intended adherence rather than actual day-to-day medication-taking behavior. The qualitative strand therefore adds contextual depth by revealing unintentional non-adherence patterns that are not fully reflected in structured adherence scales. Together, the mixed-methods findings indicate that adherence among older adults may be overestimated in surveys while still being practically compromised by memory-related and cognitive barriers.” |
| 5. Title consistency & recent evidence Ensure the title is consistent across the manuscript | Thank you for your suggestion. We have now removed hypertensive older adults with older adults living with hypertension. |
| Situate the findings within recent national data on the hypertension care cascade in Nepal (e.g. Khanal et al., medRxiv 2025) to strengthen the contextualization. Khanal A, GC S, Dahal I, Mishra S, GC VS, Wasti SP, Ghimire R. Hypertension care cascade in Nepal: findings from Nepal Demographic and Health Survey 2022. medRxiv. 2025 Mar 11:2025-03. | Thank you for this helpful suggestion. We reviewed the study by Khanal et al. based on the Nepal Demographic and Health Survey 2022. However, this work is currently available only as a preprint and is still under peer review. As journal policies, including those of PLOS ONE, are cautious regarding citation of non–peer-reviewed sources, we were uncertain about including it as a formal reference. |
| Overall, the topic and COM-B-based qualitative work are valuable, but the manuscript requires clearer handling of the null quantitative findings, limitations of the qualitative sampling, stronger mixed-methods integration, and a more cautious dementia-related framing. | Thank you for this constructive comment. We have revised the manuscript accordingly. Specifically, we have strengthened the reporting and interpretation of the null quantitative findings and clarified their implications and possible reasons (including power and measurement considerations) in the Discussion section. We have also expanded the limitations section to more clearly acknowledge the qualitative sampling constraints and their implications for transferability.  In addition, we enhanced the mixed-methods integration by more explicitly linking the quantitative and qualitative findings in the Results and Discussion sections. Finally, as suggested, we removed the dementia-related framing from the title and revised the abstract and conclusion to ensure the study implications are aligned with blood pressure control rather than dementia outcomes. |
