# Supplementary material for: Barriers and facilitators of hypertension control among older adults living with hypertension: A mixed-methods study
Source: PLOS Glob Public Health. 2026 Aug 20;6(8):e0007015. doi: 10.1371/journal.pgph.0007015 (PMC13492771; doi:10.1371/journal.pgph.0007015)
Supplement: Supplementary file 4 [file pgph.0007015.s006.docx]

Dear Editor and Reviewers,

Thank you for the careful review of our manuscript and for the opportunity to submit a revised version to PLOS Global Public Health. We sincerely appreciate the constructive feedback provided. We have carefully addressed all comments and revised the manuscript accordingly to improve its clarity and methodological transparency. Below, we provide a point-by-point response to the comments and indicate where revisions have been made in the manuscript.

Thank you again for your valuable comments and consideration.

| Reviewer’s comment | Response |
| --- | --- |
| I think that more detail is required for the Health City dataset. For example, how big is the overall dataset for Dhulikhel Municipality? Out of this, how many fulfilled the inclusion and exclusion criteria for the study? Out of this, how many participants were successfully recruited into the study? | Thank you so much for your suggestion. We have added its information in the method section from line number 117 to 125. |
