# Supplementary material for: Barriers and facilitators of hypertension control among older adults living with hypertension: A mixed-methods study
Source: PLOS Glob Public Health. 2026 Aug 20;6(8):e0007015. doi: 10.1371/journal.pgph.0007015 (PMC13492771; doi:10.1371/journal.pgph.0007015)
Supplement: Peer review history

# Peer review: Decision Letter 0

**DOI:** 10.1371/journal.pgph.0007015.r001

**Authors:** Miho Sato

7 Jan 2026

PGPH-D-25-02086

Barriers and Enablers of Hypertension Control in Nepalese Older Adults: Implications for Dementia Prevention

PLOS Global Public Health

Dear Dr. Shrestha,

Thank you for submitting your manuscript to PLOS Global Public Health. After careful consideration, we feel that it has merit but does not fully meet PLOS Global Public Health’s publication criteria as it currently stands. Therefore, we invite you to submit a revised version of the manuscript that addresses the points raised during the review process.

The reviewers recognize the importance of the topic and acknowledge the strengths of your study design, including the application of the COM-B framework and the use of a mixed-methods approach. However, several issues need to be addressed to improve the clarity, framing, and interpretation of your findings.

In particular, we ask that you:

- Clarify the study's framing in relation to dementia prevention to ensure it aligns with the scope and results;
- Strengthen the interpretation of your quantitative findings, especially where null results are reported, and discuss potential measurement and power limitations;
- Acknowledge sampling limitations in the qualitative component, particularly regarding the representativeness of identified “facilitators”;
- Elaborate on key conceptual elements of the COM-B framework to improve reader understanding;
- Integrate quantitative and qualitative findings more clearly in the discussion;
- Improve clarity, citation accuracy, and contextualization with relevant national data.

Please submit your revised manuscript by Feb 06 2026 11:59PM. If you will need more time than this to complete your revisions, please reply to this message or contact the journal office at globalpubhealth@plos.org. When you're ready to submit your revision, log on to https://www.editorialmanager.com/pgph/ and select the 'Submissions Needing Revision' folder to locate your manuscript file.

Please include the following items when submitting your revised manuscript:

- A letter that responds to each point raised by the editor and reviewer(s). You should upload this letter as a separate file labeled 'Response to Reviewers'.
- A marked-up copy of your manuscript that highlights changes made to the original version. You should upload this as a separate file labeled 'Revised Manuscript with Track Changes'.
- An unmarked version of your revised paper without tracked changes. You should upload this as a separate file labeled 'Manuscript'.

Guidelines for resubmitting your figure files are available below the reviewer comments at the end of this letter.

We look forward to receiving your revised manuscript.

Kind regards,

Miho Sato

Academic Editor

PLOS Global Public Health

Journal Requirements:

1. Please include a complete copy of PLOS’ questionnaire on inclusivity in global research in your revised manuscript. Our policy for research in this area aims to improve transparency in the reporting of research performed outside of researchers’ own country or community. The policy applies to researchers who have travelled to a different country to conduct research, research with Indigenous populations or their lands, and research on cultural artefacts. The questionnaire can also be requested at the journal’s discretion for any other submissions, even if these conditions are not met.  Please find more information on the policy and a link to download a blank copy of the questionnaire here: https://journals.plos.org/globalpublichealth/s/best-practices-in-research-reporting. Please upload a completed version of your questionnaire as Supporting Information when you resubmit your manuscript.

2. Please provide a detailed online Financial Disclosure statement. This is published with the article. It must therefore be completed in full sentences and contain the exact wording you wish to be published.

a) State the initials, alongside each funding source, of each author to receive each grant. For example: “This work was supported by the National Institutes of Health (####### to AM; ###### to CJ) and the National Science Foundation (###### to AM).”

b) State what role the funders took in the study. If the funders had no role in your study, please state: “The funders had no role in study design, data collection and analysis, decision to publish, or preparation of the manuscript.”

For more information, please go to our submission guidelines:

https://journals.plos.org/globalpublichealth/s/submission-guidelines#loc-financial-disclosure-statement

3. Please ensure that the funders and grant numbers match between the Financial Disclosure field and the Funding Information tab in your submission form. Note that the funders must be provided in the same order in both places as well.

4. Please update your online Competing Interests statement. If you have no competing interests to declare, please state: “The authors have declared that no competing interests exist.”

5. In the online submission form, you indicated that “The datasets used and/or analyzed during the current study are available from the corresponding author on reasonable request.”.

All PLOS journals now require all data underlying the findings described in their manuscript to be freely available to other researchers, either

a) In a public repository,

b) Within the manuscript itself, or

d) Uploaded as supplementary information.

This policy applies to all data except where public deposition would breach compliance with the protocol approved by your research ethics board. If your data cannot be made publicly available for ethical or legal reasons (e.g., public availability would compromise patient privacy), please explain your reasons by return email and your exemption request will be escalated to the editor for approval. Your exemption request will be handled independently and will not hold up the peer review process, but will need to be resolved should your manuscript be accepted for publication. One of the Editorial team will then be in touch if there are any issues.

For further assistance, you may go to: http://journals.plos.org/globalpublichealth/s/data-availability

6. Please ensure that the Title in your manuscript and the Title in your online submission form are the same.

7. Please provide separate main figure files in .tif or .eps format only and remove any figures embedded in your manuscript file. Please also ensure that all files are under our size limit of 10MB. Please leave the figure captions in the manuscript.

For more information about how to convert your figure files please see our guidelines: https://journals.plos.org/globalpublichealth/s/figures

8. Please ensure that you refer to Tables 3, 4 and 5 in your text as, if accepted, production will need these references to link the reader to the tables.

9. If the reviewer comments include a recommendation to cite specific previously published works, please review and evaluate these publications to determine whether they are relevant and should be cited. There is no requirement to cite these works unless the editor has indicated otherwise.

10. Please review your reference list to ensure that it is complete and correct. If you have cited papers that have been retracted, please include the rationale for doing so in the manuscript text, or remove these references and replace them with relevant current references. Any changes to the reference list should be mentioned in the rebuttal letter that accompanies your revised manuscript. If you need to cite a retracted article, indicate the article’s retracted status in the References list and also include a citation and full reference for the retraction notice.

Additional Editor Comments (if provided):

[Note: HTML markup is below. Please do not edit.]

Reviewers' comments:

Reviewer's Responses to Questions

**Comments to the Author**

1. Does this manuscript meet PLOS Global Public Health’s publication criteria?

Reviewer #1: Yes

Reviewer #2: Yes

**********

2. Has the statistical analysis been performed appropriately and rigorously?

Reviewer #1: Yes

Reviewer #2: Yes

**********

3. Have the authors made all data underlying the findings in their manuscript fully available (please refer to the Data Availability Statement at the start of the manuscript PDF file)?

The PLOS Data policy

Reviewer #1: No

Reviewer #2: Yes

**********

4. Is the manuscript presented in an intelligible fashion and written in standard English?

Reviewer #1: Yes

Reviewer #2: Yes

**********

Reviewer #1: Poor BP control, even among treated individuals, is a problem many countries in the world are currently facing, and it needs to be understood in the context of Nepal, too. Thus, this study is both relevant and essential.

However, I would like to bring your attention to the following matters-

Overall comment

The second portion of the title- “implication for dementia prevention” is not explored much in the study itself, except in the literature review section, where readers are provided with the information that high BP is a risk factor for dementia. I don’t know if that alone warrants the inclusion of the phrase in the title, when this study didn’t investigate the association or implication of such at all.

A few specific comments are below.

- Abstract conclusion- the ‘how’ of minimizing dementia risk is missing. Consider adding BP control as a pathway for minimizing dementia. For example, you could say- “Incorporating behavioral frameworks…settings like Nepal to improve BP control and therefore minimize the risk of dementia in this population.”

- Line 108-110- Please add more context about the “Healthy City Initiative dataset.” Does it include all households of the municipality? Or is it a list of people diagnosed with certain conditions residing in the city? Was the random selection done from the entire list or from a subset of the list created by filtering for selecting age and diagnosis? Who maintains the dataset, and how did the researchers access it?

This information will help the readers to understand sampling bias (if any) and understand the generalization of the findings.

- Line 130-133: It is unusual (and thus less convincing) that the researchers achieved ‘saturation’ with only 7 participants (for in-depth interviews) and two focus group discussions, and stopped their recruitment. Were additional criteria used to determine the sample size?

- Line 142- Clarify the difference between a ‘joint’ family and an ‘extended’ family.

- Line 203- The sentence is incomplete. Indicating what?

- Table 3- Two variables- type of family, and living arrangement, likely have high intercorrelation. Multicollinearity might be a concern for the multivariate regression analysis. Thus, I suggest selecting one of those variables instead of including both in the model.

- Line 182, figure 1, and Table 5- Need additional explanation on themes. Physical and psychological are straightforward, but what do ‘reflective’ and ‘automatic’ motivation mean? This will help the readers understand why ‘anger’ is ‘automatic’ motivation and others ‘reflective.’

- Line 389. Instead of participants ‘noted’, consider writing participants ‘believed’ that- because they believe that their temperament is causing high BP.

- Line 545-546, the systematic review that was cited after the sentence –“however, no significant association was found between uncontrolled blood pressure and medication adherence in this study” studied factors influencing adherence, not the association between BP and adherence. So this citation is misplaced.

- Line 552- add citation for social desirability bias as one of the explanations for non-association.

- In lines 566 to 569, the authors write- “these inconsistencies... may suggest... are more influential...” I have a problem with the “are more influential’ claim, which didn’t come from this study. Nor do the authors provide any citations for this claim. Please add citations. If not, and if it is the author's speculation only, then consider saying- “could be additional factors that influence BP outcome.”

- Line 580-584, the discussion about the influence of ‘fasting’ practice seems out of place. The authors didn’t explore fasting as a probable cause for nonadherence, nor did their qualitative findings reveal such.

- Lines 639 to 640. The authors say, “The analysis identified independent associations even after adjusting for potential confounders.” But the study didn’t find any independent (significant) association; the sentence sounds misleading. If they are talking about the benefit of using multivariate regression, perhaps they should write – “the multivariate regression approach allowed us to identify potential independent associations after adjusting for confounders.”

Reviewer #2: This manuscript addresses an important public health issue: hypertension management among older adults in a low-resource setting, and the use of the COM-B framework is a clear strength. However, several methodological and interpretive issues need revision.

1. Quantitative findings & validity

Table 3 shows no significant associations between uncontrolled hypertension and age, sex, education, BMI, comorbidity or adherence. Given existing evidence, the lack of association with BMI and adherence is implausible and likely reflects limited statistical power and/or measurement issues.

With n = 197, the multivariable model is probably underpowered. Results should be framed as “no associations detected,” with a brief post-hoc comment on power and Type II error.

The Hill-Bone scores indicate near-perfect self-reported adherence despite ~60% uncontrolled BP, suggesting strong ceiling and social desirability effects. This limitation and its implications for the null adherence–BP finding should be discussed more explicitly.

2. Qualitative sampling & “enablers”

All qualitative interviews were conducted with older adults with uncontrolled BP. This constrains the ability to identify true “enablers” of good control, as any reported facilitators have not led to control for these participants.

The manuscript should clearly acknowledge that the qualitative findings mainly reflect barriers among those with uncontrolled hypertension and that the absence of “positive deviants” is a key limitation.

3. “Dementia prevention” framing

No dementia or cognitive outcomes are analyzed, and dementia-related pathways are not examined beyond the background. The emphasis on “dementia prevention” in the title and conclusions is therefore overstated. I recommend removing “dementia prevention” from the title and tempering related claims in the abstract and Discussion.

4. Mixed-methods integration

The study is presented as mixed-methods, but the two strands are not well integrated. Suggestions: Add a clear paragraph in the Discussion that brings quantitative and qualitative results together, especially the conflicting findings, For example, qualitative data highlight “forgetfulness” as a key barrier, yet the quantitative data show very high self-reported adherence and no link between adherence and BP control

5. Title consistency & recent evidence

Ensure the title is consistent across the manuscript.

Situate the findings within recent national data on the hypertension care cascade in Nepal (e.g. Khanal et al., medRxiv 2025) to strengthen the contextualization.

Khanal A, GC S, Dahal I, Mishra S, GC VS, Wasti SP, Ghimire R. Hypertension care cascade in Nepal: findings from Nepal Demographic and Health Survey 2022. medRxiv. 2025 Mar 11:2025-03.

Overall, the topic and COM-B-based qualitative work are valuable, but the manuscript requires clearer handling of the null quantitative findings, limitations of the qualitative sampling, stronger mixed-methods integration, and a more cautious dementia-related framing.

**********

what does this mean?). If published, this will include your full peer review and any attached files.

**Do you want your identity to be public for this peer review?**  If you choose “no”, your identity will remain anonymous but your review may still be made public.

For information about this choice, including consent withdrawal, please see our Privacy Policy

Reviewer #1: No

Reviewer #2: No

**********

[NOTE: If reviewer comments were submitted as an attachment file, they will be attached to this email and accessible via the submission site. Please log into your account, locate the manuscript record, and check for the action link "View Attachments". If this link does not appear, there are no attachment files.]

Attachment Submitted filename: PGPH_Review-D-25-02086.docx

# Author comment: Author response to Decision Letter 1

**DOI:** 10.1371/journal.pgph.0007015.r002

31 Mar 2026

Attachment Submitted filename: Response to Reviewers comment.docx

# Peer review: Decision Letter 1

**DOI:** 10.1371/journal.pgph.0007015.r003

**Authors:** Kanokwan Suwannarong

15 May 2026

PGPH-D-25-02086R1

Barriers and Facilitators of Hypertension Control among older adults living with hypertension:  A Mixed-Methods Study

PLOS Global Public Health

Dear Dr. Shrestha,

Thank you for submitting your manuscript to PLOS Global Public Health. After careful consideration, we feel that it has merit but does not fully meet PLOS Global Public Health’s publication criteria as it currently stands. Therefore, we invite you to submit a revised version of the manuscript that addresses the points raised during the review process.

Kindly revise the manuscript in accordance with the reviewer’s comments.

Please submit your revised manuscript by Jun 14 2026 11:59PM. If you will need more time than this to complete your revisions, please reply to this message or contact the journal office at globalpubhealth@plos.org. When you're ready to submit your revision, log on to https://www.editorialmanager.com/pgph/ and select the 'Submissions Needing Revision' folder to locate your manuscript file.

Please include the following items when submitting your revised manuscript:

- A letter that responds to each point raised by the editor and reviewer(s). You should upload this letter as a separate file labeled 'Response to Reviewers'.
- A marked-up copy of your manuscript that highlights changes made to the original version. You should upload this as a separate file labeled 'Revised Manuscript with Track Changes'.
- An unmarked version of your revised paper without tracked changes. You should upload this as a separate file labeled 'Manuscript'.

Guidelines for resubmitting your figure files are available below the reviewer comments at the end of this letter.

As the corresponding author, your ORCID iD is verified in the submission system and will appear in the published article. PLOS supports the use of ORCID, and we encourage all coauthors to register for an ORCID iD and use it as well. Please encourage your coauthors to verify their ORCID iD within the submission system before final acceptance, as unverified ORCID iDs will not appear in the published article. *Only*  the individual author can complete the verification step; PLOS staff *cannot*  verify ORCID iDs on behalf of authors.

We look forward to receiving your revised manuscript.

Kind regards,

Kanokwan Suwannarong, Ph.D.

Academic Editor

PLOS Global Public Health

Journal Requirements:

If the reviewer comments include a recommendation to cite specific previously published works, please review and evaluate these publications to determine whether they are relevant and should be cited. There is no requirement to cite these works unless the editor has indicated otherwise.

Please review your reference list to ensure that it is complete and correct. If you have cited papers that have been retracted, please include the rationale for doing so in the manuscript text, or remove these references and replace them with relevant current references. Any changes to the reference list should be mentioned in the rebuttal letter that accompanies your revised manuscript. If you need to cite a retracted article, indicate the article’s retracted status in the References list and also include a citation and full reference for the retraction notice.

[Note: HTML markup is below. Please do not edit.]

Reviewers' comments:

Reviewer's Responses to Questions

**Comments to the Author**

Reviewer #2: All comments have been addressed

Reviewer #3: (No Response)

**********

publication criteria?

Reviewer #2: Yes

Reviewer #3: Yes

**********

3. Has the statistical analysis been performed appropriately and rigorously?

Reviewer #2: Yes

Reviewer #3: Yes

**********

4. Have the authors made all data underlying the findings in their manuscript fully available (please refer to the Data Availability Statement at the start of the manuscript PDF file)?

The PLOS Data policy

Reviewer #2: Yes

Reviewer #3: Yes

**********

5. Is the manuscript presented in an intelligible fashion and written in standard English?

Reviewer #2: Yes

Reviewer #3: Yes

**********

Reviewer #2: (No Response)

Reviewer #3: I notice that the authors have incorporated feedback from the previous round of peer review.

I have only one comment - I think that more detail is required for the Health City dataset. For example, how big is the overall dataset for Dhulikhel Municipality? Out of this, how many fulfilled the inclusion and exclusion criteria for the study? Out of this, how many participants were successfully recruited into the study?

**********

what does this mean?). If published, this will include your full peer review and any attached files.

**Do you want your identity to be public for this peer review?**  If you choose “no”, your identity will remain anonymous but your review may still be made public.

For information about this choice, including consent withdrawal, please see our Privacy Policy

Reviewer #2: **Yes:** Ashok Khanal

Reviewer #3: No

**********

[NOTE: If reviewer comments were submitted as an attachment file, they will be attached to this email and accessible via the submission site. Please log into your account, locate the manuscript record, and check for the action link "View Attachments". If this link does not appear, there are no attachment files.]

# Author comment: Author response to Decision Letter 2

**DOI:** 10.1371/journal.pgph.0007015.r004

25 May 2026

Attachment Submitted filename: Reviewers comment_Second.docx

# Editor report: Decision Letter 2

**DOI:** 10.1371/journal.pgph.0007015.r005

**Authors:** Julia Robinson

20 Jul 2026

Barriers and Facilitators of Hypertension Control among older adults living with hypertension:  A Mixed-Methods Study

PGPH-D-25-02086R2

Dear Mrs Shrestha,

We are pleased to inform you that your manuscript 'Barriers and Facilitators of Hypertension Control among older adults living with hypertension:  A Mixed-Methods Study' has been provisionally accepted for publication in PLOS Global Public Health.

Before your manuscript can be formally accepted you will need to complete some formatting changes, which you will receive in a follow up email. A member of our team will be in touch with a set of requests.

Please note that your manuscript will not be scheduled for publication until you have made the required changes, so a swift response is appreciated.

IMPORTANT: The editorial review process is now complete. PLOS will only permit corrections to spelling, formatting or significant scientific errors from this point onwards. Requests for major changes, or any which affect the scientific understanding of your work, will cause delays to the publication date of your manuscript.

If your institution or institutions have a press office, please notify them about your upcoming paper to help maximize its impact. If they'll be preparing press materials, please inform our press team as soon as possible -- no later than 48 hours after receiving the formal acceptance. Your manuscript will remain under strict press embargo until 2 pm Eastern Time on the date of publication. For more information, please contact globalpubhealth@plos.org.

Thank you again for supporting Open Access publishing; we are looking forward to publishing your work in PLOS Global Public Health.

Best regards,

Julia Robinson

Executive Editor

PLOS Global Public Health

***********************************************************

Reviewer Comments (if any, and for reference):
